# Melanocyte-Fibroblast Crosstalk Under Visible Light and Infrared-A Exposure: A Narrative Review of Implications for Photoaging and Dyschromia

**DOI:** 10.7759/cureus.115511

**Published:** 2026-08-31

**Authors:** Aylin Kerime Rojas López, Carlos Alejandro Arragan Lezama, Arismendy Yohen Maradiaga Irias, Haizel Valencia Romero, José Luis de Jesús Arguello Hernández, Julio César Flores Rodríguez

**Affiliations:** 1 Internal Medicine, Hospital General de Culiacán "Dr. Bernardo J. Gastélum", Culiacán, MEX; 2 Outpatient Care, Universidad Anáhuac Xalapa, Veracruz, MEX; 3 Institute of Dermatology, Universidad ORT, Montevideo, URY; 4 Integrative and Regenerative Medicine, Universidad Nacional Autónoma de México, Mexico City, MEX; 5 Primary Health Care, Universidad Anáhuac Xalapa, Xalapa, MEX; 6 Aesthetic and Regenerative Medicine, Punto Bell, Monterrey, MEX; 7 Aesthetic and Regenerative Medicine, Sociedad Mexicana de Investigación en Medicina Estética (SOMIME), Monterrey, MEX

**Keywords:** crosstalk, dermal photoaging, dyschromia, fibroblast activation, infrared-a, melanocyte, visible light

## Abstract

Solar radiation extends beyond the ultraviolet spectrum to include visible light and infrared-A wavelengths, which have traditionally been considered biologically inert. Emerging evidence indicates that these longer wavelengths act both independently and in combination to influence communication between skin cells, contributing meaningfully to photoaging and pigmentary changes. This narrative review synthesizes existing peer-reviewed research on how visible light and infrared-A radiation alter signaling between dermal and epidermal cells and how these alterations may underlie disorders of pigmentation and structural skin aging. A structured search of PubMed and Scopus (January 2016-June 2026) was conducted to identify English-language, peer-reviewed studies examining the mechanisms by which these wavelengths affect skin cell biology and intercellular communication, encompassing in vitro, ex vivo, animal, and clinical research. The synthesized findings indicate that visible light and infrared radiation jointly influence cellular signaling pathways that govern pigment production, breakdown of the skin's structural matrix, and cellular aging processes. Accumulating, predominantly preclinical evidence suggests that these wavelengths should no longer be overlooked in the study of skin biology's response to sunlight. Accumulating evidence points to distinct and overlapping mechanisms by which each spectral range promotes cellular aging, structural matrix changes, and disruption of the signaling molecules that dermal cells use to regulate pigment-producing cells in the skin's outer layer. This disruption alters key communication factors that drive overactivation of pigment cells, resulting in uneven pigmentation. A separate mechanism, independent of this cell-to-cell communication pathway, involves direct activation of pigment-producing cells by visible light through a distinct cellular signaling cascade. These findings suggest that broadening photoprotection beyond the ultraviolet range may be a reasonable strategy, while also identifying candidate, largely investigational therapeutic targets for addressing photoaging and pigmentary disorders that require further clinical study.

## Introduction and background

Human skin is the body's largest organ and is directly and continuously exposed to the full electromagnetic spectrum of solar radiation. The photoaging and carcinogenic effects of the ultraviolet radiation (UVR; 100-400 nm) component emitted from the sun's radiation have been extensively characterized, but the remaining portion of the spectrum above 400 nm (visible light (VL) spectrum) and far during the night spectrum (infrared-A (IRA); 760-1400 nm) contribute together around 90% of the solar radiation at the earth's surface [1]. Historically, these longer wavelengths were considered less biologically relevant than UVR, although some effects have been recognized for decades; more recent mechanistic and clinical studies have shown that both VL and IRA can induce reactive oxygen species (ROS) formation, extracellular matrix (ECM) remodeling, and melanogenic activation in human skin [2,3].

Intrinsically driven aging is the normal process, which is done chronologically. Photodamaged or photoaging of skin is the premature aging of skin, caused by this chronic cumulative solar radiation that clinically results in wrinkling, loss of elasticity, “broken skin” (telangiectasias), and dyspigmentation [1,4]. UVB and UVA are still the most important individual photodamaging agents for DNA damage and for degrading collagen, respectively, but the synergic action of VL and IRA in photoaging is gaining more and more recognition as being clinically relevant, particularly in darker skin types (Fitzpatrick IV-VI) in which the hyperpigmentation caused by VL exposure is more evident and likely to persist longer than that caused by UVA1 [4].

Skin functions as a dynamic cellular ecosystem in which dermal fibroblasts and epidermal melanocytes are thought to continuously signal to one another in a bidirectional paracrine relationship [5]. Fibroblasts produce and release the various melanogenic growth factors and cytokines such as stem cell factor (SCF), hepatocyte growth factor (HGF), basic fibroblast growth factor (bFGF), Dickkopf-1 (DKK1), and neuregulin-1 (NRG-1), as well as transforming growth factor-beta (TGF-β), that act upon melanocytes via specific receptors to affect melanocyte proliferation, differentiation, melanin production, and dendricity [6]. In return, melanocytes have the ability to prevent the radiation-mediated oxidative injury to the fibroblasts by sequestration of ROS with melanin deposition. Disruption of this homeostatic paracrine network under chronic exposure to solar radiation is now recognized as being responsible for the many pigmentary disorders and dermal aging phenotypes [7].

Pigmentation disorders are important conditions worldwide, being more prevalent in dermatological conditions such as hyperpigmented disorders - including dyschromia [8], melasma, solar lentiginous, post-inflammatory hyperpigmentation, and periorbital hyperpigmentation - in high sunlight exposure and high melanin populations. Evidence that VL (particularly the blue-violet waveband, 400-450 nm) induces more persistent hyperpigmentation in darker skin types than UVA1 and that IRA exposure is associated with fibroblast senescence and ECM degradation suggests potential implications for the clinical management of these conditions and for broader-spectrum photoprotection strategies [4,9]. This review aims to summarize the mechanistic evidence published since 2016 on melanocyte-fibroblast paracrine crosstalk induced by VL and IRA and to outline its implications for photoaging and dyschromia.

## Review

Methodology

This is a narrative review structured to improve transparency of the literature search; it does not follow a formal systematic review methodology (e.g., PRISMA). Two electronic databases, PubMed and Scopus, were searched for English-language peer-reviewed articles published between 1 January 2016 and 1 June 2026. Boolean searching of the search terms included the following: ("visible light" OR "infrared-A" OR "near-infrared") AND ("melanocyte" OR "fibroblast") AND ("paracrine crosstalk" OR "photoaging" OR "dyschromia" OR "melasma" OR "hyperpigmentation" OR "cellular senescence" OR "SASP" OR "SCF" OR "HGF" OR "DKK1" OR "MITF" OR "melanogenesis" OR "opsin"). The primary search strategy was complemented by hand-searching lists of key articles and reviews to find further relevant studies not indicated by the search. The search strings used for each specific database are provided in the appendix.

Inclusion criteria comprised the following: (i) peer-reviewed studies in English; (ii) published between 1 January 2016 and 1 June 2026; (iii) either in vitro, in vivo, or clinical studies whose primary subject matter was the effect of VL on melanocytes, fibroblasts, and/or on the paracrine interactions between melanocytes and fibroblasts; and (iv) narrative or systematic review providing a comprehensive overview of the effects of VL and/or IRA on the melanocyte-fibroblast interactions.

Studies were excluded if they were conference abstracts without an available full text, preprints, editorials or commentaries without original data, case reports, or non-peer-reviewed grey literature (e.g., theses). Titles and abstracts were screened (AKRL, CAAL) for relevance to melanocyte-fibroblast crosstalk following VL/IRA exposure; discrepancies were resolved by discussion with a third author (AYMI). Full texts of potentially eligible articles were then reviewed by the author team to confirm eligibility. The evidence was synthesized using a narrative approach.

Synthesis of evidence

Photobiology of VL and IRA Light in Human Skin

VL wavelengths span the color spectrum from 400 nm (violet) to 700 nm (red) and penetrate to a depth corresponding to each wavelength length, with the shorter wavelengths ending up mostly in the epidermis and the longer ones in the deeper layers of the dermis [10]. VL interacts with key chromophores such as melanin, flavins, porphyrins, and nitric oxide and with several recently described opsins, which are found in non-ocular pigmented cells [3]. Recently, it has been found that the G-protein-coupled photoreceptor Opsin 3 (OPN3) is a major blue-light melanogenesis inducer in melanocytes, particularly in darker skin phototypes [11]. Following activation by blue light, OPN3 allows calcium influx, which then results in the activation of calcium/calmodulin-dependent protein kinases (CaMKII) [11], which go on to phosphorylate and upregulate tyrosinase and dopachrome tautomerase (DCT), the rate-limiting enzymes involved in melanin synthesis, and the mitogen-activated protein kinases (MAPKs) and extracellular-signaling-regulated kinases (ERKs) [11-13].

Of special note, an additional study from 2024 to 2025 confirmed that under the action of blue light, TRPV1 is activated after OPN3, and a newly defined blue-light pigmentation pathway exists: OPN3-TRPV1-calcium, which not only activates clusterin but also inhibits melanocyte autophagy and promotes the nuclear localization of PAX3, a transcription factor involved in melanogenesis [13]. VL affects melanocyte pigmentation through both autocrine mechanisms (direct activation of the melanocyte itself) and paracrine mechanisms, whereby blue-light-exposed keratinocytes release factors that upregulate tyrosinase and DCT in neighboring melanocytes [14].

IRA-Penetration Depth and Biological Targets

This infrared radiation, termed IRA (760-1400 nm), can easily go deeper into the dermis and directly influence the dermal fibroblasts, vascular endothelial cells, and subcutaneous adipocytes [9]. In mammalian cells, cytochrome c oxidase (COX), a component of the mitochondrial electron transport chain, is considered one of the principal intracellular photoreceptors/targets of IRA radiation [15]. However, exposure to low-dose IRA at acute exposures triggers the photobiomodulatory effects by stimulation of the absorption of IRA by COX, resulting in a transient increase of mitochondrial membrane potential and ATP synthesis [3,16]. In vitro studies using chronic IRA irradiation protocols designed to mimic cumulative daily solar exposure have demonstrated continuous oxidative stress in cultured human dermal fibroblasts, characterized by prolonged inhibition of cell proliferation, increased intracellular ROS, mitochondrial dysfunction, and morphological changes typical of premature cellular senescence [9]. These findings are derived from cell-culture models and have not yet been confirmed in vivo in human skin.

In an in vitro study, Yang et al. (2022) treated cultured human dermal fibroblasts with low, non-thermal doses of IRA radiation and observed significant cellular damage despite the absence of a measurable thermal effect, including activation of apoptotic pathways, increased ROS production, and morphological changes consistent with senescence [9]. While derived from an experimental cell-culture model, these findings raise the clinically relevant hypothesis that the absence of perceptible heat during IRA exposure does not indicate the absence of photobiological damage - a hypothesis that warrants confirmation in human skin studies.

Additional Impact: Synergistic and Accumulative Effects

In addition to their individual effects, VL and IRA appear to act synergistically with UVR to amplify skin photodamage. In one ex vivo human skin study, VL, UVA, and UVB contributed approximately 50%, 46%, and 4%, respectively, to total measured cutaneous ROS generation under the specific irradiation protocol used, challenging the traditional UVB-centric view of photodamage [7]. These proportions reflect a single experimental irradiation protocol and should not be generalized to natural, unfiltered sunlight exposure without further validation. The oxidative and inflammatory stress produced by the two stresses, VL and IRA, in combination, in addition to the effect of UVR, is much larger than the oxidative and inflammatory stress described by each alone [16,17]. This is consistent with a comparative in vitro study using physiologically relevant doses of individual and combined UV, visible, and infrared solar-simulated light on matched human dermal fibroblasts and keratinocytes, which found measurable ROS generation and both mitochondrial and nuclear DNA damage under combined exposure [18,19].

Fibroblast responses to the VL and IRA

ECM Remodeling

The dermal fibroblasts play a pivotal role in the production of the constituents of skin ECM, such as types 1 and III collagen, elastin, and fibronectin, as well as regulating the turnover of these proteins by secreting matrix metalloproteinases (MMPs) [17]. Fibroblasts exposed to VL and IRA have been found to have increased expression of MMP-1 (interstitial collagenase) and decreased expression of procollagen I, consistent with the decrease in ECM in photoaged human skin in both in vitro and ex vivo studies [18]. The experiments on the pathway level suggest that IRA stimulates the MAP kinase (MAPK)/AP-1 pathway so that MMP-1 is increased and collagen is fragmented in fibroblasts while simultaneously reducing the expression of the fibrillin-1, fibrillin-2, and elastin genes [2,18], but this has yet to have been directly confirmed in human skin biopsies after irradiation.

Of interest, UV and VL appear to induce overlapping ROS-mediated oxidative signaling pathways in fibroblasts, with similar upregulation of MMP-1 and MMP-9 [7]. In vitro studies have found comparable effects of VL and IRA exposure on ECM-related gene expression, including MMP1 and MMP3 [18]. Proposed mechanisms linking ROS generation to VL-induced fibroblast damage remain experimentally derived and have not been confirmed in human skin.

Fibroblast Senescence and Senescence-Associated Secretory Phenotype (SASP) ​​​Induction

The irreversible cell-cycle arrest that occurs in dermal fibroblasts as part of cellular senescence is increasingly known as being caused by both intrinsic aging and cumulative photodamage, which lead to the pro-inflammatory SASP [20]. UV radiation is still the most well-established inducer of fibroblast senescence in human skin; the experimental in vitro studies have also revealed that IRA could induce senescence, but only through mitochondrial oxidative damage, mainly in cultured human fibroblasts, and not in vivo [9]. Based on in vitro and ex vivo tissue studies, it has been shown that senescent fibroblasts exhibit an altered secretion pattern that includes an increase in SCF, HGF, IL-1α, IL-1β, IL-6, and TNF-α and a decrease in DKK1, WIF-1, and SDF-1 in photoaged dermis [20,21]. The pro-melanogenic effects of each of these mediators (SCF, HGF, and GDF15) on melanocyte c-KIT/MITF as well as MET/PI3K-AKT signaling are described in the Paracrine Mediators section below.

Wnt Signaling Dysregulation

An important signaling pathway that is involved in melanocyte regulation is the Wnt/β-catenin pathway that regulates the expression of MITF and the activity of melanogenic enzymes [21]. Fibroblasts are an important source of Wnt tone (both ligand and antagonist) within the skin microenvironment due to their secretion of both Wnt ligands as well as secreted frizzled-related proteins (sFRP) and DKK1, Wnt antagonists [6]. The in vitro and ex vivo studies show that, under photodamaged conditions (IRA, UVR, and VL exposure), resident fibroblasts downregulate DKK1 and WIF-1 production, thus removing the inhibitory effect exerted by them on the Wnt/β-catenin pathway in neighboring melanocytes and inducing MITF-supported melanogenesis [21,22]. In vitro evidence suggests that senescent fibroblasts secrete sFRP2, which paradoxically activates non-canonical NF-κB and β-catenin signaling in melanocytes [23]; this finding requires confirmation in photodamaged human skin. Altered fibroblast secretion of Wnt modulators has been proposed as an important mechanism linking VL and UV exposure to acquired hyperpigmentation disorders [23], although this remains to be confirmed in photodamaged human skin.

Melanocytes are stimulated by VL and IRA light

Direct Melanogenic Activation by VL

VL activates melanocytes directly in various ways (photoreceptor-dependent and oxidative). The OPN3-mediated pathway described above is mainly activated by blue-violet wavelength light (415-450 nm) and is especially high in melanocytes of dark skin races [11,13]. However, in the absence of OPN3, red light and IRA activate COX in melanocytes' mitochondria, which leads to an increase in second messengers and melanogenesis too [3,16]. A prior narrative review summarizing in vitro mechanistic data reported distinct effects of red, near-infrared (NIR), green, and blue-violet (BIV) light on melanocyte biology: red/NIR light appeared protective via mitochondrial photobiomodulation, green light stimulated melanin production without UVR-like DNA damage, and BIV light upregulated tyrosinase, producing more persistent pigmentation than other visible wavelengths, a finding also supported by controlled human exposure data [10]. As with other narrative-review sources cited here, these conclusions reflect the primary literature summarized therein rather than independent critical appraisal by the present authors.

In controlled human exposure studies it has been shown that hyperpigmentation of skin induced by VL is different clinically - it persists for longer than that induced by ultraviolet light (UVB/UVA) (weeks after a single exposure vs. days for UVB/UVA tanning); occurs more strongly in Fitzpatrick phototypes III-VI; and it is not blocked effectively by the conventional mineral-free UVB/UVA sunscreens [4,8]. This evidence base is not based on in vitro models but on human volunteer irradiation studies, which are directly clinically relevant.

Melanocyte Senescence

In vitro and ex vivo studies show that melanocytes can also become photoinduced senescent, paradoxically retaining high-amplitude melanogenic activity while losing replicative capacity [24]. Senescent melanocytes have been shown experimentally to produce their own SASP, including the chemokine IP-10 (CXCL10) [25], which can act as an autocrine factor in melanocytes by binding to CXCR3 to keep the melanocytes senescent and also as a paracrine factor on neighboring keratinocytes and dermal fibroblasts to induce senescence [24]. This VL/IRA-associated paracrine spread of senescence may establish a self-perpetuating feedback loop, in which photodamaged melanocytes impair fibroblast function while senescent fibroblasts, in turn, secrete pro-melanogenic signals [24]. In addition, senescence melanocytes produce SASP that negatively affects the surrounding cells by encouraging mitochondrial ROS and CXCR3 signaling, causing disruption of keratinocyte proliferation and contributing to epidermal thinning [20].

Stimulatory Effects of Endothelin and SCF Axes

A second pathway important in the activation of melanocytes by solar radiation is the endothelin-1 (ET-1) pathway. Experimental evidence indicates that solar radiation (via a UV-mediated mechanism) promotes endothelin-1 binding to endothelin receptor B (EDNRB) on melanocytes, activating the MAPK signaling pathway and driving melanogenesis [25]; direct evidence for VL/IRA-specific activation of this axis in human skin remains limited. The epidermal melanocytes in photoaged skin are subjected to repeated paracrine melanogenic stimuli by activated fibroblasts that secrete increased quantities of ET-1 as part of their activated SASP pool, under the influence of chronic exposure to UV and infrared light [8,25]. It has also been proposed, based on evidence that dermal mast cells degranulate in response to thermal, physical, and radiation stimuli, that IRA-activated mast cells release histamine and other bioactive mediators capable of stimulating melanocyte proliferation via H₂ receptors, potentially contributing to an inflammatory melanogenic circuit in IRA-exposed skin [26]; direct experimental confirmation specific to IRA-induced mast cell activation in skin is currently limited.

Paracrine mediators of melanocyte-fibroblast crosstalk under VL and IRA

This section reviews the key paracrine mediators of melanocyte-fibroblast crosstalk described in the literature over the past decade, including the SCF/c-KIT axis, HGF/MET signaling, DKK1's role in hypopigmented zones, neuregulin-1 and phototype-specific effects, TGF-β-mediated anti-melanogenic regulation, and the convergence of these pathways at the level of intracellular signaling.

SCF and the c-KIT Axis

A paracrine mediator that is most uniform in its expression from fibroblasts to damaged skin melanocytes is SCF, a ligand for a tyrosine kinase receptor called c-KIT. UVR is chronic radiation that induces activation of the dermal compartment with increased upregulation of SCF secretion and activation of c-KIT on melanocytes, leading to MAPK/ERK and PI3K/AKT pathways with, lastly, increased phosphorylation of MITF causing increased tyrosinase activity [8,21]. Clinical tissue studies have shown that SCF is consistently upregulated in the dermis of melasma lesions compared with adjacent normal skin, and epidermal SCF expression has been proposed as a marker associated with melanocyte hyperactivation [8]. In vitro data indicate that IRA-senescent fibroblasts secrete more SCF than growth-arrested (non-senescent) fibroblasts, offering a proposed mechanistic - though not yet clinically confirmed - link between IRA-induced fibroblast senescence and acquired dyschromia [21].

HGF and MET Signaling

As to melanocytic activity, dermal fibroblasts express and secrete HGF, which binds to the MET receptor on melanocytes and stimulates melanocytic proliferation, migration, and melanin production [6,27]. To boot, the photodamaged fibroblasts produce HGF as part of their SASP, which in turn could cause melanocyte hyperplasia and hyperpigmentation. Strikingly, however, there is paradoxically increased HGF secretion in the fibroblasts of the vitiligo patch where the homeostasis is disturbed, also alongside increased DKK1, which has been shown to decrease E-cadherin in melanocytes and decrease their adhesion to keratinocytes, making them prone to loss [27]. This highlights the notion that the action of the same paracrine factors can be pro- or anti-melanocytic in different circumstances; this is an important consideration for the array of dyschromic responses to solar radiation.

DKK1 as a Regulator of Hypopigmented Skin Zones

Palmoplantar skin and other non-sun-exposed areas are relatively hypopigmented, in part because of DKK1, a secreted Wnt-signaling inhibitor produced mostly by fibroblasts, which normally suppresses melanocyte activity [6]. In photodamaged skin, fibroblasts appear to downregulate DKK1 production, relieving its inhibitory effect on melanogenesis and permitting increased MITF activity via the Wnt/β-catenin pathway [22]. Under normal physiologic conditions, it is hoped that the levels of DKK1 will balance some of the functions of some of the target genes (downstream), such as melanocyte-specific genes [24,26,27]. In vitiligo, selected fibroblasts, however, may paradoxically produce excess DKK1 that inhibits the activity of melanocytes and the activity of melanocyte-specific genes [28]. One mechanistic explanation for the predilection of acquired hyperpigmentation disorders for a photodistributed pattern is the differential regulation of DKK1, which is presumably induced by radiation between sun-exposed and non-exposed skin [27,28].

Neuregulin-1 (NRG-1) and Skin-Type-Specific Melanogenesis

NRG-1 is secreted by fibroblasts of darker skin types and utilizes its ErbB3/ErbB4 receptor in melanocytes to induce melanogenesis, which may be one of the reasons darker skin has a heightened melanin level [8]. NRG-1 upregulation in genetically susceptible individuals following VL/IRA exposure has been inferred from in vitro and phototype-specific mechanistic data rather than demonstrated directly in irradiated human skin and may help explain persistent pigmentation in darker skin types. This pathway underlines the need to consider individual genomic and microenvironmental factors to investigate the clinical significance of melanocyte-fibroblast crosstalk in VL and IRA [8].

The Balance of Anti-Melanogenic to TGF-β

Fibroblasts secrete TGF-β, which has a normal anti-melanogenic and anti-proliferative effect on melanocytes [6]. Chronic UVA and IRA exposure also negatively regulates melanocyte activity by dampening the inhibition of melanocyte activity provided by the TGF-β/Smad pathways in fibroblasts in a negative fashion by reducing TGF-β production [2]. The simultaneous upregulation of pro-melanogenic mediators (SCF, HGF) and downregulation of the anti-melanogenic mediator TGF-β under photodamage suggests a coordinated, mutually reinforcing shift that may help sustain hyperpigmentation.

Integration of molecular signaling pathways

The paracrine mediators described above converge on a limited set of intracellular signaling cascades in melanocytes. c-KIT, MET, EDNRB, and OPN3-TRPV1 receptor activation all feed into the MAPK/ERK cascade, phosphorylating MITF and driving tyrosinase-dependent melanin synthesis (detailed in the Photobiology and Direct Melanogenic Activation sections above) [9-11,13,21]. In parallel, HGF/MET and SCF/c-KIT signaling activate PI3K/AKT, supporting melanocyte survival and proliferation [6,8]. Fibroblast-derived Wnt modulators (DKK1, WIF-1, sFRP2, and GDF15) converge on the Wnt/β-catenin pathway, as described in the Wnt Signaling Dysregulation section, to regulate nuclear MITF transcription [21-23]. Finally, IRA-induced ROS activates NF-κB in fibroblasts, sustaining the SASP and chronic low-grade inflammation ("inflammaging") described in the Fibroblast Senescence and SASP section, while simultaneously suppressing DKK1 expression and further shifting the paracrine balance toward melanocyte stimulation [17,20]. Collectively, these convergent, largely preclinically characterized pathways are proposed to jointly drive MITF activation, tyrosinase upregulation, and persistent pigmentation, as summarized in the integrated model (Table 1).

**Table 1:** Summary of the Evidence Supporting Visible Light- and Infrared-A-Induced Melanocyte-Fibroblast Signaling. Note: The table is generated by the authors to present findings in a logical way, using and citing references [2,6-8,9,11,13,15-22,24,26-33]. Visible light (VL); infrared-A (IRA); opsin 3 (OPN3); transient receptor potential vanilloid 1 (TRPV1); calcium/calmodulin-dependent protein kinase II (CaMKII); microphthalmia-associated transcription factor (MITF); calcium ion (Ca²⁺); dopachrome tautomerase (DCT); paired box 3 (PAX3); cytochrome c oxidase (COX); reactive oxygen species (ROS); adenosine triphosphate (ATP); ultraviolet radiation (UVR); ultraviolet A (UVA); ultraviolet B (UVB); extracellular matrix (ECM); matrix metalloproteinase 1 (MMP-1); activator protein 1 (AP-1); mitogen-activated protein kinase (MAPK); Jun proto-oncogene transcription factor (c-Jun); senescence-associated secretory phenotype (SASP); stem cell factor (SCF); hepatocyte growth factor (HGF); interleukin-1 alpha/beta (IL-1α/β); interleukin-6 (IL-6); tumor necrosis factor-alpha (TNF-α); Dickkopf-related protein 1 (DKK1); Wnt inhibitory factor 1 (WIF-1); stromal cell-derived factor 1 (SDF-1); KIT proto-oncogene receptor tyrosine kinase (c-KIT); MET proto-oncogene receptor tyrosine kinase (MET); phosphoinositide 3-kinase (PI3K); protein kinase B (AKT); wingless/integrated signaling pathway (Wnt); neuregulin-1 (NRG-1); erythroblastic leukemia viral oncogene homolog 3 (ErbB3); erythroblastic leukemia viral oncogene homolog 4 (ErbB4); transforming growth factor-beta (TGF-β); mothers against decapentaplegic homolog (Smad); nuclear factor kappa-light-chain-enhancer of activated B cells (NF-κB); endothelin-1 (ET-1); endothelin receptor type B (EDNRB); histamine H2 receptor (H2R); interferon gamma-induced protein 10 (IP-10); C-X-C chemokine receptor type 3 (CXCR3).

Mechanism/Pathway	Predominant Evidence Type	Model/Clinical Context	Main Findings	Clinical Interpretation	Evidence Maturity
OPN3/TRPV1/CaMKII/MITF activation (blue-violet light)	Experimental (cell culture) + controlled human exposure	Cultured melanocytes; human skin phototype studies (III–VI)	Blue light activates OPN3, triggering Ca²⁺ influx, CaMKII activation, tyrosinase/DCT upregulation, and MITF-driven melanogenesis; TRPV1 co-activation inhibits autophagy and promotes PAX3 nuclear localization [11,13]	Direct, fibroblast-independent pigmentation mechanism; supports iron-oxide/blue-light-blocking photoprotection, particularly in darker phototypes	Preclinical (in vitro) + controlled human exposure
IRA–mitochondrial COX/ROS signaling	Experimental (in vitro)	Cultured human dermal fibroblasts	Acute low-dose IRA transiently boosts ATP via COX (photobiomodulation); chronic IRA causes sustained oxidative stress, mitochondrial dysfunction, and senescence-like morphology [9,15,16]	Biologically plausible driver of fibroblast senescence and photoaging; not yet confirmed in vivo in human skin	Preclinical (in vitro)
VL/IRA/UVR-induced ROS generation (comparative)	Experimental (ex vivo human skin)	Ex vivo human skin explants	VL contributes ~50% of total cutaneous ROS, exceeding UVA (46%) and UVB (4%) [7]	Challenges UVB-centric photoprotection paradigms; supports full-spectrum sunscreen strategies	Preclinical (Ex vitro)
ECM remodeling (MMP-1/AP-1, procollagen suppression)	Experimental (in vitro)	Cultured dermal fibroblasts	VL and IRA increase MMP-1 and decrease procollagen I, fibrillin-1/2, and elastin gene expression via MAPK/AP-1 and c-Jun activation [2,18,19]	Mechanistic basis for VL/IRA-associated structural photoaging; supports antioxidant and botanical photoprotectant use	Preclinical (in vitro)
Fibroblast senescence and SASP induction	Experimental (in vitro), limited ex vivo tissue data	Cultured/irradiated human dermal fibroblasts	IRA and UV induce irreversible cell-cycle arrest with SASP secretion (↑SCF, HGF, IL-1α/β, IL-6, TNF-α; ↓DKK1, WIF-1, SDF-1) [9,20,21]	Establishes senescent fibroblasts as a chronic pro-melanogenic paracrine source; underlies proposed senolytic strategies (investigational)	Preclinical (in vitro), limited ex vivo
SCF/c-KIT axis	Clinical tissue studies + in vitro	Melasma lesional vs. perilesional skin; senescent fibroblast cultures	Dermal SCF upregulated in melasma lesions; IRA-senescent fibroblasts secrete more SCF than growth-arrested controls, activating melanocyte c-KIT/MITF [8,19,21]	Directly links fibroblast senescence to acquired dyschromia; candidate investigational therapeutic target	Clinical tissue + preclinical (in vitro)
HGF/MET axis	Experimental (in vitro), context-dependent (vitiligo vs. photoaging)	Cultured fibroblasts; vitiligo and photoaged skin comparisons	HGF from senescent fibroblasts promotes melanocyte proliferation/motility via MET–PI3K/AKT; it is paradoxically elevated (with DKK1) in vitiligo, reducing melanocyte adhesion [6,8,27]	Illustrates context-dependent (pro- vs. anti-melanocytic) paracrine signaling; complicates therapeutic targeting	Preclinical (in vitro)
DKK1/Wnt–β-catenin regulation	Experimental (in vitro) + mechanistic tissue data	Sun-exposed vs. non-exposed skin: vitiligo skin	Photodamage suppresses fibroblast DKK1/WIF-1, releasing Wnt/β-catenin inhibition and increasing MITF activity; DKK1 paradoxically elevated in vitiligo [6,22,24,26-28]	Explains photodistributed pattern of hyperpigmentation; supports investigational topical DKK1 augmentation	Preclinical (in vitro) + mechanistic tissue data
NRG-1/ErbB3/ErbB4 axis	Experimental (in vitro), genetic/phototype-specific	Fibroblasts from darker skin phototypes	NRG-1 secretion and ErbB3/ErbB4 signaling upregulated in genetically susceptible individuals under VL/IRA exposure [8]	Highlights phototype- and genotype-specific susceptibility to persistent pigmentation	Preclinical (in vitro), genetic/phototype-specific
TGF-β/Smad suppression	Experimental (in vitro)	Cultured fibroblasts under UVA/IRA exposure	Chronic UVA/IRA exposure reduces fibroblast TGF-β production, releasing its normal anti-melanogenic/anti-proliferative restraint on melanocytes [2,6]	Converges with SCF/HGF upregulation to reinforce persistent hyperpigmentation	Preclinical (in vitro)
NF-κB/inflammaging loop	Experimental (in vitro)	Cultured senescent fibroblasts	IRA-induced ROS activates NF-κB, sustaining SASP cytokine/MMP expression and suppressing DKK1, shifting paracrine balance toward melanogenesis [17,20]	Frames chronic inflammation as a self-perpetuating amplifier of photoaging-associated dyschromia	Preclinical (in vitro)
Endothelin-1/EDNRB and mast cell–histamine/H2R signaling	Experimental (in vitro/ex vivo)	UV/IRA-exposed skin and dermal mast cells	Solar radiation activates ET-1/EDNRB–MAPK signaling in melanocytes; IRA-activated mast cells release histamine, stimulating melanocyte proliferation via H₂ receptors [25,26]	Identifies additional non-fibroblast paracrine and inflammatory routes to melanocyte activation	Preclinical (in vitro/ex vivo)
Melanocyte senescence and IP-10/CXCR3 signaling	Experimental (in vitro/ex vivo)	Senescent melanocyte cultures	Senescent melanocytes retain high melanogenic activity but lose replicative capacity; IP-10/CXCR3 signaling propagates senescence to neighboring keratinocytes and fibroblasts [24,25]	Supports a bidirectional, self-reinforcing senescence–pigmentation cycle between melanocytes and fibroblasts	Preclinical (in vitro/ex vivo)
VL-blocking photoprotection (iron oxide, tinted sunscreens)	Clinical/interventional (comparative sunscreen studies)	Human volunteer sunscreen efficacy studies	Iron oxide-containing sunscreens reduce melasma recurrence and outperform non-tinted formulations in blocking VL-induced pigmentation; efficacy varies by formulation, not just iron oxide concentration [18,29-33]	Directly actionable clinical recommendation; supports full-spectrum photoprotection guidelines	Clinical/interventional

Collectively, the direct cellular response of cells to both VL and IRA, as well as fibroblast senescence and matrix remodeling in a manner that is dependent upon time of exposure, will stimulate fibroblasts to produce paracrine signals. Ultimately, these signals will activate the microphthalmia-associated transcription factor (MITF), which increases tyrosinase activity, thereby increasing melanin production and ultimately creating persistent pigmentation. The cell-specific and intercellular mechanisms involved in this process have been illustrated in an integrated model depicted in Figure 1.

**Figure 1:**
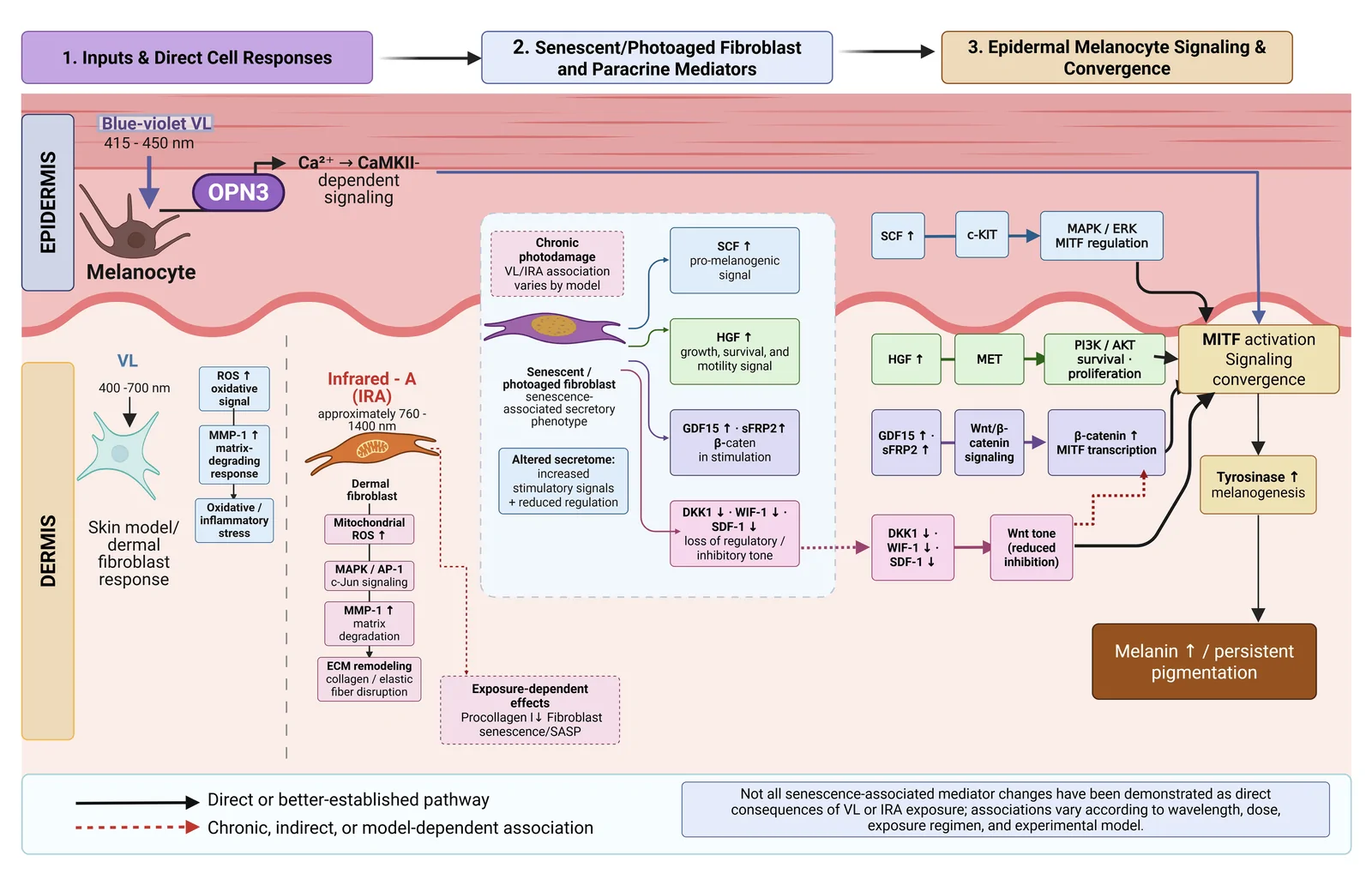
Proposed integrated model of selected visible light- and infrared-A-associated fibroblast-melanocyte signaling in photodamaged skin. Visible light (VL); infrared-A (IRA); opsin 3 (OPN3); calcium ion (Ca²⁺); calcium/calmodulin-dependent protein kinase II (CaMKII); reactive oxygen species (ROS); matrix metalloproteinase 1 (MMP-1); mitogen-activated protein kinase (MAPK); activator protein 1 (AP-1); Jun proto-oncogene transcription factor (c-Jun); extracellular matrix (ECM); senescence-associated secretory phenotype (SASP); stem cell factor (SCF); hepatocyte growth factor (HGF); growth differentiation factor 15 (GDF15); secreted frizzled-related protein 2 (sFRP2); Dickkopf-related protein 1 (DKK1); Wnt inhibitory factor 1 (WIF-1); stromal cell-derived factor 1 (SDF-1); KIT proto-oncogene receptor tyrosine kinase (c-KIT); MET proto-oncogene receptor tyrosine kinase (MET); extracellular signal-regulated kinase (ERK); phosphoinositide 3-kinase (PI3K); protein kinase B (AKT); Wingless-related integration site (Wnt); and microphthalmia-associated transcription factor (MITF). Solid arrows indicate direct or better-established pathways, whereas dashed arrows indicate chronic, indirect, exposure-dependent, or model-dependent associations. Created in BioRender. Flores Rodríguez, J. C. (2026) https://BioRender.com/48oe7a5

Clinical implications

Melasma and Solar Lentigines

Melasma is now understood to involve more than melanocyte hyperactivation alone. It reflects broader dysregulation of the skin's cellular microenvironment, encompassing dermal fibroblasts, mast cells, vascular endothelial cells, and the basement membrane [8,29]. The molecular evidence reviewed here firmly positions VL and IRA as significant contributors to melasma pathogenesis through fibroblast-mediated pro-melanogenic paracrine signaling. This is supported clinically by the observation that adding iron oxide (a blue-VL absorber) to broad-spectrum UVB-UVA sunscreens reduces melasma recurrence, confirming the pathogenic role of VL [18]. Similarly, solar lentigines - a hallmark of photoaged hyperpigmentation - are hypothesized, based on converging mechanistic and observational tissue evidence, to arise through the compounded, long-term effects of VL and IRA on fibroblast senescence and perilesional SCF upregulation [30-32]; this remains a proposed rather than definitively established causal pathway.

Photoprotection Implications

Current photoprotection guidelines and sunscreen testing standards predominantly focus on UVB (SPF) and, to a lesser extent, UVA protection. The evidence that VL and IRA each contribute substantially to both photoaging and dyschromia urgently demands expansion of these frameworks [4,30]. Based on the reviewed evidence, broad-spectrum photoprotection strategies with varying levels of supporting evidence include iron oxide pigments for VL (particularly blue-violet, 415-450 nm) blockade - supported by clinical/interventional sunscreen studies; antioxidant formulations targeting IRA-generated ROS (e.g., vitamin C, vitamin E, idebenone) - currently supported mainly by in vitro and mechanistic data; botanical photoprotectants (e.g., *Polypodium leucotomos*), which have shown efficacy in preclinical models in preventing VL- and IRA-induced MMP-1 upregulation and changes in fibrillin-1, fibrillin-2, and elastin [2] - clinical confirmation specific to VL/IRA protection is limited; and topical antioxidant formulations proposed to attenuate IRA-mediated mitochondrial ROS in dermal fibroblasts - currently investigational.

Physical and behavioral approaches are as important as chemical-based methods when it comes to protecting your body from UV radiation. By utilizing items such as wide-brimmed hats, sunglasses, sunscreen on unprotected areas, and clothing that protects you from UV radiation, you will have a multi-layered approach to protect yourself from harmful rays. The use of multiple layers for protection is beneficial because if one layer fails, another can help protect you from the rest of the harmful effects [33]. Sunscreen provides some degree of protection, but there are other products available that can offer even greater protection. Tinted sunscreens are examples of this. They work because they contain colorants (iron oxide), which provide an extra level of protection. This does not mean that all tinted sunscreens are created equal or offer the same amount of protection. For example, a study comparing the effectiveness of several tinted sunscreens to non-tinted sunscreens found that three of four tinted sunscreens were significantly better at blocking the UV-A and UV-B radiation that causes pigmentation [34]. One of those studies compared two different tinted sunscreens; one with a higher concentration of iron oxide was no better than the lower concentration [35]. Currently, there is evidence that indicates tinted sunscreens limit damage caused by VL and may help prevent or manage melasma relapse. There is, however, currently very little research available regarding the standardization of testing criteria for measuring the effectiveness of VL-blocking agents or the optimal shade range for consumers. As a result, selecting a product should be done based upon its demonstrated ability to block VL and an acceptable shade rather than simply choosing a "tinted" product [34].

Therapeutic Targets

The fibroblast-melanocyte paracrine axis suggests several candidate, investigational therapeutic targets for dyschromia: (i) c-KIT/SCF inhibition is hypothesized to reduce fibroblast-driven melanogenic drive in melasma and solar lentigines, based on preclinical data [8,32]; (ii) MET/HGF antagonism is proposed to reduce melanocyte proliferation and motility driven by senescent fibroblasts; this is mechanistically inferred only [6]; (iii) Wnt modulation (topical DKK1 augmentation or sFRP2 inhibition) - a preclinically supported concept for restoring anti-melanogenic Wnt tone in photodamaged skin [22,23]; (iv) Senolytic strategies are hypothesized to reduce pro-melanogenic SASP signaling. Based on senescence biology, they have not yet been tested clinically in photoaging [17,28]; and (v) MITF transcriptional regulation - targeting MITF activity downstream of OPN3/TRPV1-CaMKII signaling - is currently supported only by upstream mechanistic evidence [13,36].

Although these pathways represent plausible therapeutic targets biologically, most rely mainly on mechanistic evidence or preclinical results. Clinical efficacy, safety, selectivity, and long-term effects have not yet been adequately established. Therefore, approaches involving SCF/c-kit, HGF/MET signaling, modulation of Wnt signaling, or senolytics should be considered investigational and need further validation before they can be recommended for routine clinical use.

Research gaps and future directions

Despite substantial progress, several critical knowledge gaps persist. First, the vast majority of studies examining VL and IRA effects on melanocyte-fibroblast crosstalk have been conducted in monolayer in vitro systems, which fail to recapitulate the three-dimensional ECM environment, basement membrane, and immune cell context of living skin [1,5]. Three-dimensional skin equivalent models and organotypic cultures incorporating both fibroblasts and melanocytes under physiologically relevant irradiation protocols would be valuable for advancing this field. Second, the majority of studies have focused on a limited number of phototypes, and robust mechanistic data from diverse global skin phototypes - particularly types V and VI - remain scarce, despite these populations bearing the highest burden of VL-induced dyschromia [4,30]. Third, the specific thresholds of VL and IRA dose, wavelength, and exposure duration required to induce clinically meaningful melanocyte-fibroblast crosstalk alterations are poorly defined, and defining these thresholds will be critical for setting evidence-based photoprotection standards [9]. Fourth, the interplay between VL/IRA-induced fibroblast senescence and immune senescence in the photoaged dermis - particularly the role of dermal macrophages and mast cells in amplifying melanogenic paracrine signals - represents a largely unexplored frontier [37]. Finally, clinical trials evaluating interventions specifically targeting the fibroblast-melanocyte axis (senolytics, anti-SCF biologics, topical DKK1 modulators) in photoinduced dyschromia have not yet been conducted and represent a high-priority translational research need [29].

## Conclusions

Accumulating mechanistic evidence, much of it preclinical, indicates that VL and IRA radiation play a more significant role in skin photobiology than previously recognized. A convergent body of molecular evidence published since 2020 establishes that both spectral domains independently and synergistically drive fibroblast senescence, ECM remodeling, and profound dysregulation of the paracrine secretome through which dermal fibroblasts govern epidermal melanocyte behavior. The key paracrine mediators of this crosstalk - SCF, HGF, DKK1, NRG-1, GDF15, and TGF-β - are all quantitatively and qualitatively altered by VL and IRA exposure, creating a dermal microenvironment persistently permissive to melanocyte hyperactivation and dyschromia. The direct activation of melanocytes by VL via the OPN3/TRPV1/CaMKII/MITF axis provides an additional, fibroblast-independent mechanism of pigmentary perturbation. Collectively, these findings support reconsideration of UV-centric photoprotection paradigms in favor of full-spectrum strategies addressing the complete solar emission reaching human skin and highlight a potential frontier of therapeutic targets within the melanocyte-fibroblast axis for managing photoaging and dyschromia, pending further clinical validation.

## Appendices

Appendix A

**Table 2:** Search Strings Used for Each Database With Limiters.

Databases	Search Strings
PubMed	("visible light"[Title/Abstract] OR "visible light radiation"[Title/Abstract] OR "infrared-A"[Title/Abstract] OR "infrared A"[Title/Abstract] OR "near-infrared"[Title/Abstract] OR "IRA"[Title/Abstract]) AND ("melanocyte"[Title/Abstract] OR "melanocytes"[Title/Abstract] OR "melanogenesis"[Title/Abstract] OR "fibroblast"[Title/Abstract] OR "fibroblasts"[Title/Abstract] OR "dermal fibroblast"[Title/Abstract]) AND ("paracrine"[Title/Abstract] OR "crosstalk"[Title/Abstract] OR "cell signaling"[Title/Abstract] OR "photoaging"[Title/Abstract] OR "photoageing"[Title/Abstract] OR "dyschromia"[Title/Abstract] OR "melasma"[Title/Abstract] OR "hyperpigmentation"[Title/Abstract] OR "cellular senescence"[Title/Abstract] OR "SASP"[Title/Abstract] OR "SCF"[Title/Abstract] OR "stem cell factor"[Title/Abstract] OR "HGF"[Title/Abstract] OR "hepatocyte growth factor"[Title/Abstract] OR "DKK1"[Title/Abstract] OR "Dickkopf"[Title/Abstract] OR "MITF"[Title/Abstract] OR "opsin"[Title/Abstract] OR "OPN3"[Title/Abstract]) Filters: English language; Publication date from 2016/01/01 to 2026/06/01
Scopus	(TITLE-ABS-KEY ("visible light" OR "infrared-A" OR "infrared A" OR "near-infrared" OR "IRA" ) AND TITLE-ABS-KEY ( "melanocyte" OR "melanocytes" OR "melanogenesis" OR "fibroblast" OR "fibroblasts" OR "dermal fibroblast" ) AND TITLE-ABS-KEY ("paracrine" OR "crosstalk" OR "photoaging" OR "photoageing" OR "dyschromia" OR "melasma" OR "hyperpigmentation" OR "cellular senescence" OR "SASP" OR "SCF" OR "stem cell factor" OR "HGF" OR "hepatocyte growth factor" OR "DKK1" OR "Dickkopf" OR "MITF" OR "opsin" OR "OPN3" ) ) AND PUBYEAR > 2015 AND PUBYEAR < 2027 AND ( LIMIT-TO ( LANGUAGE , "English" ) ) AND ( LIMIT-TO ( DOCTYPE , "ar" ) OR LIMIT-TO ( DOCTYPE , "re" ) )
